# Supplementary material for: The Antiangiogenic Effect of VEGF-A siRNA-FAM-Loaded Exosomes
Source: Bioengineering (Basel). 2025 Aug 26;12(9):919. doi: 10.3390/bioengineering12090919 (PMC12467468; doi:10.3390/bioengineering12090919)
Supplement: Supplementary file 1 [file bioengineering-12-00919-s001.zip › bioengineering-3753636-supplementary.pdf]

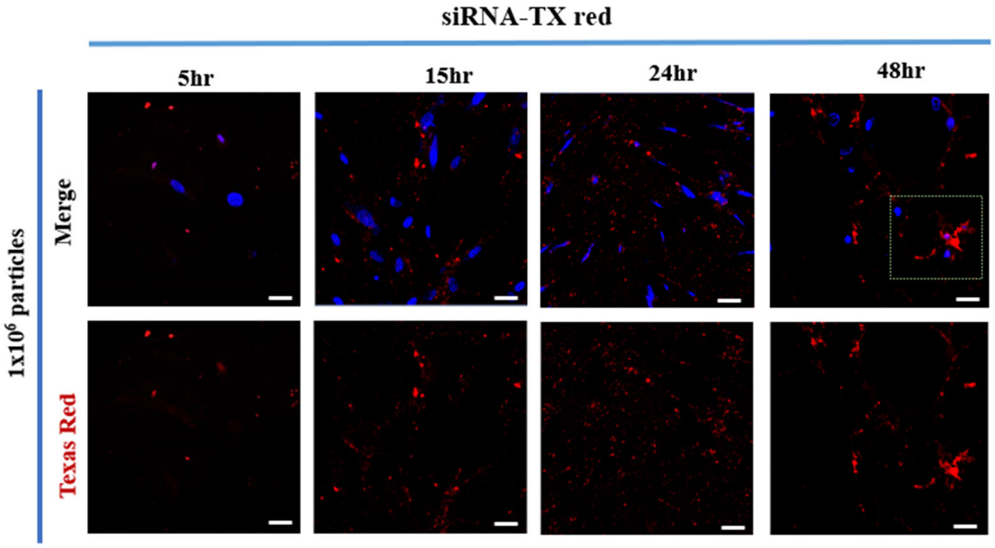

Figure S1. Loading VEGFA siRNA-FAM into exosomes. A transfection efficiency test was first performed using siRNA-TX Red. Using a positive control siRNA (TX-Red), the research team directly transfected purified exosomes and achieved high transfection efficiency. Arrows indicated that the cytosol of the target cells was labeled red by exosomes loaded with siRNA-TX at 48 h post-treatment, as observed under confocal microscopy. The scale bar represents 200 μm.
